# miR-370 regulates cell proliferation and migration by targeting EGFR in gastric cancer

**DOI:** 10.3892/or.2022.8409

**Published:** 2022-09-15

**Authors:** Tao Ning, Haiyang Zhang, Xinyi Wang, Shuang Li, Le Zhang, Ting Deng, Likun Zhou, Rui Liu, Xia Wang, Ming Bai, Shaohua Ge, Hongli Li, Dingzhi Huang, Guoguang Ying, Yi Ba

Oncol Rep 38: 384–392, 2017; DOI: 10.3892/or.2017.5660

Subsequently to the publication of the above paper, the authors have drawn to the Editors' attention that an error was made during the assembly of Fig. 2C. Essentially, the image selected to represent the PCDNA EGFR group was erroneously selected from those for the mimics NC group. This error arose inadvertently as a consequence of multiple original pictures being opened simultaneously during the process of collating the data.

The corrected version of Fig. 2 is shown on the next page. Note that the revisions made to this figure do not affect either the results or the conclusions reported in the paper. The authors are grateful to the Editor of Oncology Reports for allowing them the opportunity to publish this Corrigendum, and apologize to the readership for any inconvenience caused.

## Figures and Tables

**Figure 2.**
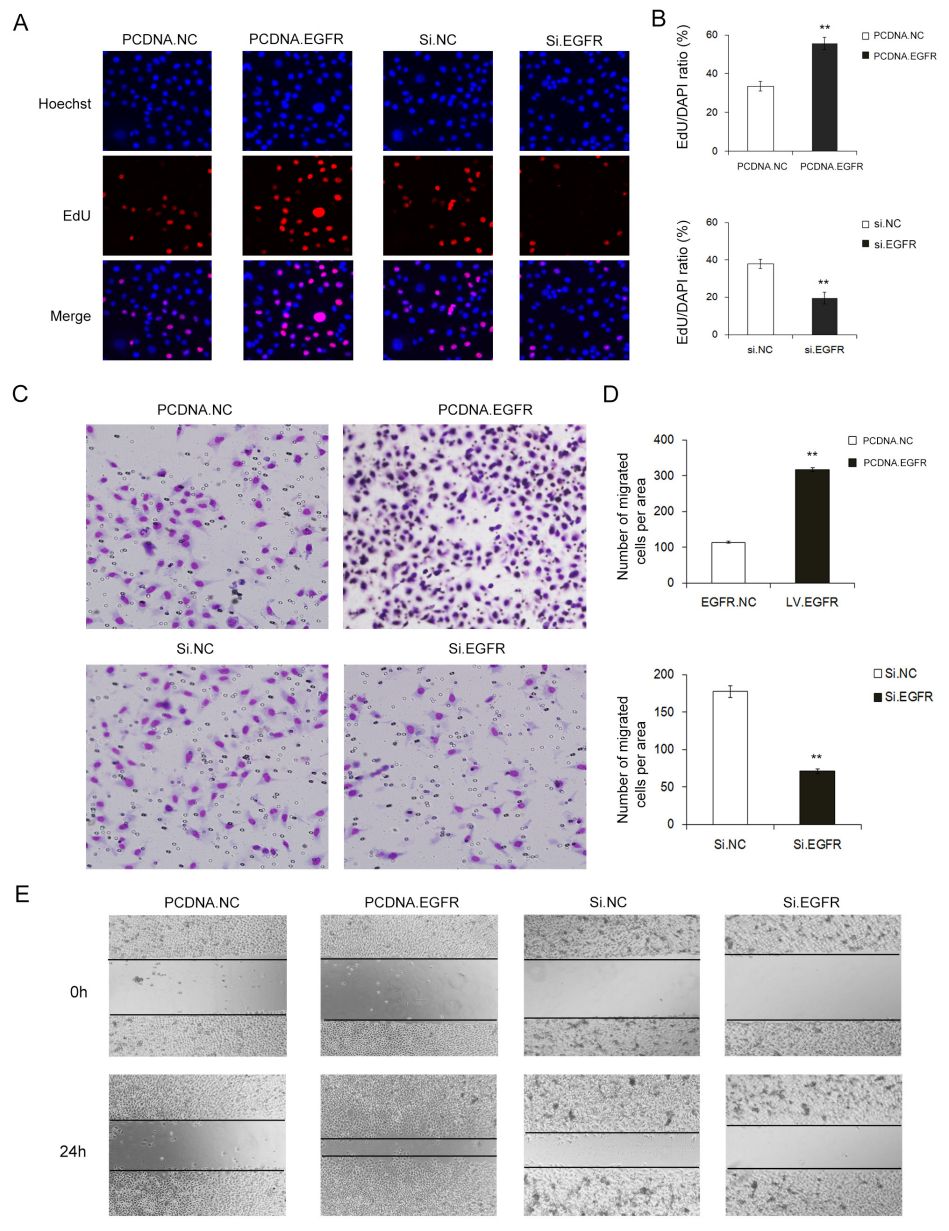
Effect of EGFR overexpression or silencing on the cell proliferation and migration in GC cells. (A) EdU assay demonstrated that overexpression of EGFR promoted cell proliferation, whereas knockdown of EGFR inhibited cell proliferation. (B) Quantitative analysis of A (n=3). (C) Transwell assay showed that overexpression of EGFR enhanced cell migration, whereas silencing of EGFR inhibited cell migration. (D) Quantitative analysis of C (n=3). (E) Evaluation of EGFR-mediated cell migration using wound healing assay. PCDNA NC refers to the control empty plasmid, PCDNA EGFR refers to the overexpression plasmid, si NC refers to control scrambled siRNA, and Si-2 EGFR refers to EGFR siRNA-2; ^**^P<0.01.

